# Non-Coding RNAs Steering the Senescence-Related Progress, Properties, and Application of Mesenchymal Stem Cells

**DOI:** 10.3389/fcell.2021.650431

**Published:** 2021-03-19

**Authors:** Jingyi Cai, Hexu Qi, Ke Yao, Yang Yao, Dian Jing, Wen Liao, Zhihe Zhao

**Affiliations:** ^1^State Key Laboratory of Oral Diseases and National Clinical Research Center for Oral Diseases, Department of Orthodontics, West China Hospital of Stomatology, Sichuan University, Chengdu, China; ^2^State Key Laboratory of Oral Diseases and National Clinical Research Center for Oral Diseases, Department of Implantology, West China Hospital of Stomatology, Sichuan University, Chengdu, China; ^3^Department of Orthodontics, Osaka Dental University, Hirakata, Japan

**Keywords:** mesenchymal stem cells, senescence, non-coding RNA, extracellular vesicles, molecular therapy

## Abstract

The thirst to postpone and even reverse aging progress has never been quenched after all these decades. Unequivocally, mesenchymal stem cells (MSCs), with extraordinary abilities such as self-renewal and multi-directional differentiation, deserve the limelight in this topic. Though having several affable clinical traits, MSCs going through senescence would, on one hand, contribute to age-related diseases and, on the other hand, lead to compromised or even counterproductive therapeutical outcomes. Notably, increasing evidence suggests that non-coding RNAs (ncRNAs) could invigorate various regulatory processes. With even a slight dip or an uptick of expression, ncRNAs would make a dent in or even overturn cellular fate. Thereby, a systematic illustration of ncRNAs identified so far to steer MSCs during senescence is axiomatically an urgent need. In this review, we introduce the general properties and mechanisms of senescence and its relationship with MSCs and illustrate the ncRNAs playing a role in the cellular senescence of MSCs. It is then followed by the elucidation of ncRNAs embodied in extracellular vesicles connecting senescent MSCs with other cells and diversified processes in and beyond the skeletal system. Last, we provide a glimpse into the clinical methodologies of ncRNA-based therapies in MSC-related fields. Hopefully, the intricate relationship between senescence and MSCs will be revealed one day and our work could be a crucial stepping-stone toward that future.

## Introduction

Mesenchymal stem/stromal cells (MSCs), representing one subpopulation of adult stem cells derived from mesoderm lineage characterized by surface markers such as CD105 and CD44, are sources of mesodermal derivatives like chondrocytes, osteocytes, and adipocytes (Uccelli et al., 2008). MSCs *in vivo* contribute to the maintenance and regulation of related organs’ homeostasis, while maneuvered modification or transplantation of MSCs appears to be a prior remedy in diversified pathological processes (Galipeau and Sensébé, 2018). Specifically, the role of MSCs as the lifelong reservoir of several cell types and its counteractive properties on inflammation and other pathological processes account for the therapeutic effect of MSC-related strategies. Additionally, characteristics like their wide accessibility from bone marrow, periosteum, adipose tissues, and umbilical cord blood; relatively simplified cultivation and expansion condition; and immuno-evasive properties accelerate its utilization as both autologous and allogeneic therapies in acute and chronic diseases. Once administered, the modified MSCs would function via proliferation and differentiation to replenish damaged tissue and by paracrine actions to modulate different responses (Uccelli et al., 2008; English, 2013; Moretta et al., 2015; Neri, 2019). However, MSCs *in vivo* from aged donors or *in vitro* from late passage cultivation display a significant reduction in immunomodulation, generation potential, and homing ability (Turinetto et al., 2016; de Witte et al., 2017; Ganguly et al., 2017; Loisel et al., 2017). Senescence-associated secretory phenotype (SASP) and modified transcription profile in senescent MSCs were also reported (Özcan et al., 2016; Mieczkowska et al., 2018). Besides the possibility of treatment failure, aged MSCs may even exacerbate pathological process and deteriorate the situation (Szychlinska et al., 2017; Zhai et al., 2019). Consequently, all these promises and pitfalls accentuate the significance of the exploration and clarification of molecular conducting the aging process of MSCs.

Aging, as a gradual and inevitable process, culminates with impaired capacities of both self-repair and homeostasis maintenance of tissues (Schultz and Sinclair, 2016; Ermolaeva et al., 2018). Among diversified phenotypes observed in aging process, exhaustion and impaired activity of stem cells stands out as a hot spot of research (Ermolaeva et al., 2018). The influences of aging on stem cell functionality are multi-layered and intricate. Age-related changes in the systemic context and microenvironment as well as the intrinsic aging as cellular senescence (CS) would all dent their effective regeneration and other capacities. To elaborate a bit on that, MSCs locate in specific areas, termed niches, where they generally keep a state of quietness to minimize metabolic or replicative damage. Once sensing the demands and changes of milieu, they would turn to generate functional progeny or secrete related factors to exert paracrine effects, which implies their vulnerability to systematic changes. On the other hand, CS, a state of irreversible cell cycle arrest, would compromise the self-renewal and differentiation ability, as well as change the niche to induce integral senescence (Rando, 2006).

Notably, advances achieved in the sequencing technique have translated the research of RNAs from dream to reality, which unveils a new, complex, and attractive regulation network (Cakouros and Gronthos, 2020; Rossi and Gorospe, 2020; Sui et al., 2020). Non-coding RNAs (ncRNAs), a collective term for molecules like microRNAs (miRNAs), long non-coding RNAs (lncRNAs), and circular RNAs (circRNAs), originally viewed as the transcriptional noise produced by mistakes or artificial operation, are now appreciated as the main regulators of life processes. Different expression profiles of miRNAs between the centenarian group (successful aging) and the normal group were identified, and ncRNA-dependent strategies for age-related diseases (ARD) diagnosis and treatments were exploited (Serna et al., 2012; Lai et al., 2019). Though the detailed panorama of MSC senescence is far from complete, the roles of ncRNAs, as an epigenetic drift with a potent effect, sparked numerous pieces of research in recent years (Ali et al., 2016; Kilpinen et al., 2016; Kim et al., 2017; Gomez-Verjan et al., 2018; Cai et al., 2019; Puvvula, 2019; Bravo et al., 2020; Cakouros and Gronthos, 2020; Rossi and Gorospe, 2020; Sui et al., 2020).

In this review, we present an illustrated map of senescence as well as its interplay with MSCs, then focus on the reported ncRNA regulation of the senescent process in MSCs. Secondly, we report extracellular vesicles’ (EVs’) function as cargo to deliver ncRNAs as age-related messengers in MSCs. Last, we discuss the potential fields that hold great promise for the clinical application of MSCs with ncRNA regulation.

## Causes, Features, Molecular Mechanism of CS, and Its Relation With MSCs

Cellular senescence is deemed as both the main hallmark and the causative factor in the aging process. In eukaryotic life, cells would exit cell cycle permanently under specific situations stimulated by factors like telomere shortening, irreversible DNA damage, and oncogenic mutations, as well as metabolic and oxidative stress. According to different stimuli, consensus is generally achieved on the three main categories of CS: replicative senescence, oncogene-induced, and stress-induced senescence, while the last two can also be termed premature senescence (Pignolo et al., 2019; Neri and Borzì, 2020).

Replicative senescence is characterized by an initiation activity as telomere shortening. Linear chromosomes include a group of specialized structures called telomeres at the end of the DNA sequences, which is composed of repeats of tandem sequence TTAGGG and shelterin complex. Telomere acts as a protective structure to DNA damage but shortens progressively with each cellular division due to the end-replication flaw. Once the replication activity reaches the “Hayflick limit,” the DNA end would be finally exposed and elicit a DNA damage response (DDR), which would consequently lead to a cascade reaction resulting in CS (Bernal and Tusell, 2018). Though the shortening of telomeres seems to be irreversible and destined, cells also develop a system to slow down degradation. Specifically, enzyme telomerase would step in to maintain the hemostasis of telomeres, cushioning DNA damage during replication. The telomerase reverse transcriptase (TERT) and the telomeric repeat template as telomerase RNA (TERC) are two major components of telomerase, and their down-regulation in CS would exacerbate the degradation of telomere (Rossi and Gorospe, 2020). For instance, the TERT gene was reported to be targeted by miR-195, and artificial deletion of miR-195 or abrogation of age-induced miR-195 would rejuvenate MSCs as well as reverse the senescence clock (Okada et al., 2016). On the other side, telomere shortening is not an absolute prerequisite for CS to occur. Oncogene-induced senescence (OIS) is conducted by the imbalance between a set of oncogenic signals and tumor suppressors. The theory of OIS contributes to burgeoning researches into CS as a therapeutic strategy to cancer. Last, stress-induced senescence is a collective name for the CS caused by different intrinsic or environmental stress like excessive reactive oxygen species (ROS), which is often related to abnormal mitochondrial activities and metabolic disorders (Yuan et al., 2020). It is worth noting that oncogene and stress-induced senescence would also elicit telomere damage and DDR. Meanwhile, endogenous and exogenous stimuli often function simultaneously to induce CS. It can be concluded that factors induce CS in a collaborative way rather than in an independent manner.

**FIGURE 1:**
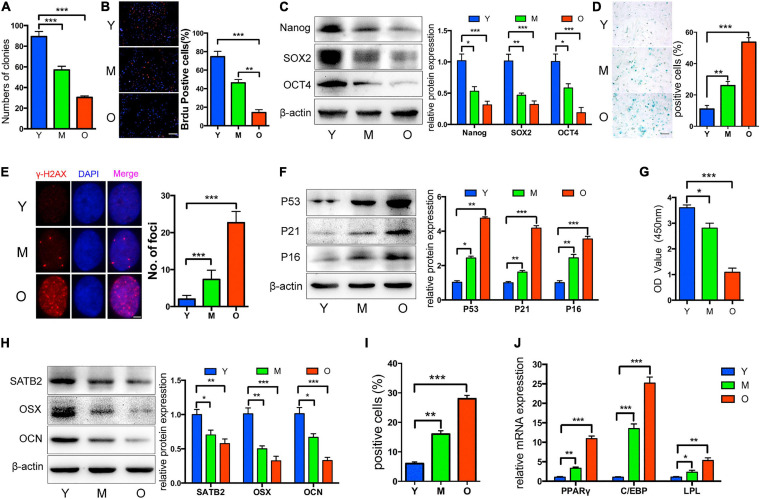
Properties of MSCs from aged rats. Compared to Y and M groups, O group was characterized by: **(A)** Decreased colony forming units; **(B)** reduced ability of proliferation; **(C)** decreased Nanog, SOX2, and OCT4 protein levels; **(D,E)** more senescent cells, as indicated by SA-β-gal staining and cH2AX foci formation; **(F)** upregulated P53, P21, and P16; **(G)** reduced osteogenic differentiation indicated by Alizarin red staining; **(H)** decreased SATB2, OSX, and OCN; **(I,J)** increased adipogenic ability detected by oil red O staining, and higher mRNA levels of adipogenic markers. Y, M, O stand as young, middle-age, and old rat group. *p < 0.05, **p < 0.01, ***p < 0.001. Scale bars: 100 lm **(B)**; 100 lm **(D)**; 4 lm **(E)**. Reprinted from Xu et al. (2018).

Once turned into the senescence state, cells would display several common and symbolic properties that can be utilized for identification. To begin with, morphological changes such as larger sizes and flattened outlooks are confirmed. DNA damage foci, senescence-associated heterochromatic foci (SAHF), and other epigenetic modifications occur to suppress pro-proliferative gene expression, thus promoting permanent cell cycle arrest. In contrast to its definition as “aged” cells, senescent cells still display active metabolism activity and some may develop SASP to modulate the microenvironment, which may be causative for age-related disorders. R. Xu et al. have exploited the common properties of bone marrow mesenchymal stem cells (BM-MSCs) from aged rats, as indicators of CS in MSCs (Xu et al., 2018; Figure 1). Typically, senescence-associated beta-galactosidase (SA-β-Gal) staining and p16^INK4a^ expression testing are two classic markers for CS detection (Zhao et al., 2017; Zhai et al., 2019; Yuan et al., 2020). In MSCs, specialized markers of aging, such as MSC-EVs containing miR-146a-5p and prelamin A accumulation, were also developed (Corrigan et al., 2005; Lei et al., 2017). Interestingly, the toxic prelamin A accumulation, which contributes to the nuclear structural abnormalities and age-related phenotypes in MSCs, is also mechanically mediated by miRNAs. During replicative senescence, histone modification and elevated RNA polymerase II activity at the miR-141-3p promoter region activate the miR-141-3p/ZEPSTE24/prelamin A axil, resulting in the accumulation of prelamin A, which cannot successfully mature without proper cleavage mediated by ZEPSTE24 (Corrigan et al., 2005; Yu et al., 2014).

As for molecules at play, two intersected and partially exclusive processes, such as p53/p21 and/or phosphorylation of retinoblastoma protein (pRb)/p16 pathways, would account for all culminating events of senescence regardless of diversified initiation (Larsson, 2011). P53 can be activated by DDR and contribute to p21-related cell cycle arrest, which underlies the mechanism of most replicative senescence. Considerably, p53, besides its role as the key player mediating CS, controls cell death and apoptosis as well, indicating an intricate network of function. Under stress, up-regulated p53 would induce phosphatase and tensin homolog (PTEN), which would in turn suppress the two main inhibitors of p53, namely, phosphatidylinositol 3-kinase (PI3K)/protein kinase B (AKT) and silent information regulator 1 (SIRT1)/forkhead box O transcription factor 3a (FOXO3a) pathways (Hekmatimoghaddam et al., 2017). The diversified molecules and pathways involved suggest the possibility for ncRNAs like miR-34a (Williams et al., 2018) targeting SIRT1 and/or E2F family to function in CS and add to the complexity of the p53-related CS network. On the other side, the pRb/p16 pathway directly controls the cell cycle initiation, which involves the interaction between a bunch of cyclins and cyclin-dependent kinase (CDK). Normally, G1-S phase transformation requires the phosphorylation of Rb and its release from E2F to enable downstream transcription. The initial phosphorylation relies on the binding of cyclin D to CDK4/6, forming a complex to enable their nucleus location to phosphorylate Rb. Cyclin E would be up-regulated by partial de-repression of E2F at the early G1 phase and then bind to CDK2 to complete the following phosphorylation process of Rb, forming a positive feedback loop. Hyperphosphorylated pRb in late G1 phase could eventually release from the E2F, activating the transcription activities that are essential for cell cycle transition. As for the inhibitory factors, the INK4 family, including p16^INK4a^ binding CDK4/6, and the CIP/KIP family represented by p27^KIP1^ and p21^CIP1^ binding CDK2/4/6 at different phases, could all block the activation of downstream molecules like E2F, thereby contributing to most premature senescence (Goel et al., 2018). Once again, interplay and concurrent activation of two pathways as well as the independent function could be observed in CS (Beauséjour et al., 2003; Rando, 2006; Fafián-Labora et al., 2019; Yuan et al., 2020). Strikingly, miR-17-92 cluster was proved to be able to assist cells to re-enter the cell cycle via both p53/p21 and pRb/p16 pathways. MiR-17-92 directly targets and suppresses Rb2/p130, and promotes PI3K/AKT pathway by down-regulating PTEN and targeting p21^WAF^ to repress p53–p21 signaling (Hekmatimoghaddam et al., 2017). Intriguingly, the E2F could induce the expression of this miRNA cluster in turn, which builds a negative feedback loop between E2F and miR-17-92 cluster (Dolezalova et al., 2012). MiR-29c-3p would also promote the senescence of human MSCs (hMSCs) in both pathways as reported in the latest research (Shang et al., 2016). Other than the two classical mechanisms, Wnt/β-catenin, tumor growth factor-β (TGF-β)/bone morphogenetic protein receptors (BMPs)/Smads, and mitogen-activated protein kinase (MAPK) pathways are also demonstrated to mediate senescence progression, which would be discussed later (Teufel and Hartmann, 2019). A blueprint of the crucial molecules and pathways in CS suggests that relevant ncRNAs may merit our attention for strategies to retard the aging process or ARD.

Unequivocally, CS favors homeostasis maintenance and tumor suppression to some extent. However, its retardation and blocking effects on physiological processes and properties are rather unwanted for clinic use. In particular, like all stem cells, MSCs are sensitive to niche alternation and have their function severely impaired when going through CS (Costa et al., 2020). Once senescent, most MSCs would display common features like compromised ability of osteogenesis and homing, as well as different secretome profiles, which account for their undesirable side effects. First, a halt in cell cycle would impair the regeneration of MSCs to a great extent. Aged MSCs show a higher inclination for adipocyte differentiation at the cost of inhibited osteogenesis (Maredziak et al., 2016), which may be explained by the elevated activity of peroxisome proliferator-activated receptor γ (PPAR-γ) while miR-188 (Wu et al., 2014), miR-196a (Candini et al., 2015), and lncRNA Bmncr (Chen et al., 2019) were verified to participate in the process (Kim and Ko, 2014). Second, the homing ability and migratory potential of MSCs to re-localize to the damaged spots to function are greatly diminished in both mice and humans (Rombouts and Ploemacher, 2003; Carlos Sepúlveda et al., 2014; Liu et al., 2017). Reduced miR-211 expression in aged hMSCs can be a case in point, postulated to cause the loss of homing capacity of cells from aged donors via signal transducers and activators of transcription 3 (STAT3)/miR-211/STAT5A (Hu et al., 2016). Last, age-related changes in molecular composition and extracellular matrix (ECM) structure of the senescent milieu were reported to even outweigh the intrinsic factors for senescence induction (Asumda, 2013; Wong, 2015). SASP secreted by aged MSCs would impact the niches and even contribute to pathophysiological conditions and exacerbate the aging process systematically. For example, the pro-inflammatory cytokine interleukin-6 (IL-6), as the most prominent component of SASP, would contribute to inflammation process *in vitro*, though its effect on aged MSCs as whether to exacerbate senescence or rejuvenate functionality as a response to aging remains unclear (Pricola et al., 2009; Xie et al., 2018). Likewise, insulin-like growth factor binding proteins (IGFBP) 4 and 7 could enable the senescence-inducing effect of passage 10 (P10) MSCs on P1 cells (Severino et al., 2013). All these common properties mentioned above contribute to the compromised efficiency of stem cell-based therapy, which was shown when therapeutic cells were either extracted from aged donors or administered to older recipients (Lin et al., 2019).

From another perspective, MSCs are sensitive to the environmental changes while induced senescence in MSCs can be observed along with systematic diseases like inflammation and metabolic diseases. Taking pigs with metabolic syndrome (MetS), for example, advanced senescence in MSCs from MetS animals was confirmed and miR-27b seemed to provoke the process by stimulating MAPK3 and p16, though the exact mechanism of how the background information was sensed remains to be explored (Meng et al., 2018). On the other hand, for patients suffering from diabetes mellitus, factors like high-glucose (HG) would also serve as a potent CS inducer (Yokoi et al., 2006). One study focusing on the therapeutic effect of organic nitrates on HG-induced senescence indicated that miR-130b served as the downstream factor of extracellular signal regulated kinase (ERK)/forkhead box M1 (FOXM1) pathway to enable the protective effect of nitrates on MSCs from HG-induced senescence (Xu et al., 2015). Maternal–fetal interface-derived MSCs going through aberrant CS may contribute to the pathogenesis of pre-eclampsia (PE) (Cox and Redman, 2017), which is postulated to be conducted by increased miR-495 in umbilical cord-derived MSCs in PE patients. Elevated miR-495 was reported to bind to B cell-specific moloney murine leukemia virus integration site 1 (Bmi-1) to promote the development of PE. Meanwhile, arrested cell cycle at S phase and promoted cell apoptosis appeared to be the results of miR-495 over-expression, which was worthy of further investigation (Li et al., 2017). Though the systematic elucidation of their roles in environment sensing is absent, these studies provide us with strong evidence that ncRNAs do matter in conducting environment-induced senescence, though the bigger picture is still obscure. Thus, research to map out the picture of MSC senescence, especially about the important roles played by miRNAs, lncRNAs, circRNAs, and EVs in this field, are greatly needed and will be introduced in the following sections (Table 1).

**TABLE 1:** Non-coding RNAs regulating CS of MSC.

Publish information	NcRNA	Cellular type	Expression level in senescent state	Mechanism of function
2017 (Onodera et al., 2017; Hong et al., 2020)	MiR-155-5p	BM-MSC (mice)	High	(1) Inhibit a set of antioxidants in ROS generation; (2) Target Cab39/AMPK pathway to exacerbate the mitochondrial turbulence.
2018 (Liu et al., 2018); 2015 (Zhang et al., 2015)	MiR-34a	BM-MSC (rat)	High	(1) Target at SIRT1/FOXO3a; (2) Increase ROS-related activities.
2012 (Kim et al., 2012)	MiR-486-5p	AD-MSC (human)	High	Target at SIRT1 to response to high glucose changes
2016 (Peffers et al., 2016)	MiR-199-5p	BM-MSC (human)	Low	Regulate SIRT1, TGFα, and PODXL
2015 (Liu et al., 2015)	MiR-17	BM-MSC (mice)	Low	Function via p53/miR-17/Smurf1 axil
2019 (Lee et al., 2014)	MiR-543/MiR-590-3p	MSCs (mice/human)	Low	Targeting AIMP3
2018 (Li et al., 2013)	MiR-10a	BM-MSCs (mice/human)	Low	(1) Inhibit KLF4–BAX/BCL2 pathway; (2) Induce AKT activation to hamper the apoptotic process in senescent MSCs.
2019 (Tai et al., 2020)	MiR-20b-5p; MiR-106a-5p	MSC (human)	Low	Target p21 and CCND1 to inhibit the expression of E2F1 and suppress the G1/S-phase transition of the cell cycle
2018 (Xu et al., 2018)	MiR-31a-5p	BM-MSC (rat)	High	(1) Bind E2F2 and thus recruiting SAHF foci in the nucleus; (2) Osteoblastogenesis inhibition by targeting at SATB2; (3) Osteoclastogenesis promotion by RhoA pathway.
2015 (Li et al., 2015)	MiR-188	BM-MSCs (mice/human)	High	(1) Target HDAC9 and RICTOR to promote PPARγ activity; (2) Target at a set of G1/S related Cyclin/CDKs, resulting in Rb/E2F inactivation.
2014 (Candini et al., 2015)	MiR-196a	AD-MSC (human)	High	Target HoxB7 and regulate the expression of the differentiation driver as bFGF in BMSCs.
2014 (Tomé et al., 2014)	MiR-335	BM-MSCs (human)	High	Reduce AP-1 activity
2018 (Fan et al., 2018)	MiR-1292	AD-MSC (human)	High	Target FZD4 and suppress Wnt/β-catenin
2016 (Mittal et al., 2011; Shang et al., 2016)	MiR-29c-3p	BM-MSC (human)	High	Target CNOT6 through p53–p21 and p16–pRB pathways
2016 (Hu et al., 2016)	MiR-211	BM-MSC (human)	Low	Function via STAT3/miR-211/STAT5A–ERK1/2 axil
2016 (Okada et al., 2016)	MiR-195	BM-MSC (mice)	High	Enable the telomere re-lengthening
2014 (Yu et al., 2014)	MiR-141-3p	MSC (human)	High	Function via miR-141-3p–ZMPSTE24-prelamin-A accumulation axil
2014 (Xu et al., 2015)	MiR-130b	BM-MSC (rat)	Low	Function as the downstream factor of the ERK/FOXM1 pathway
2018 (Meng et al., 2018)	MiR-27b	AD-MSC (pig)	High	Stimulate MAPK3 and p16
2017 (Li et al., 2017)	MiR-495	UCB-MSC (human)	High	Bind Bmi-1
2016 (Dimitrova et al., 2014; Xia et al., 2017)	lincRNA-p21	BM-MSC (mice)	High	(1) Scaffold to recruit hnRNP-K on p21 promoter; (2) Repress the translation/translocation of β-catenin.
2018 (Musavi et al., 2019)	Rn7SK	AD-MSC (human)	High	Scaffold to recruit p-TEFb and serves as the doorman for Pol II phosphorylation and activation
2018 (Li et al., 2018)	Bmncr	BM-MSC (mice)	Low	(1) Affect local 3D chromatin structure; (2) Upregulate FMOD level to anchor BMSCs; (3) Activate BMP2 pathway; (4) Scaffold for ABL to interact with TAZ.
2019 (Cherubini et al., 2019)	Circ-FOXP1	UCB/BM-MSC (human)	Low	Act as ceRNA sponge to miR17-3p/miR127-5p to promote non-canonical Wnt pathway and active EGFR signaling

## Micrornas, the Makeup Artist of Genomics in Senescent MSCs

**FIGURE 2:**
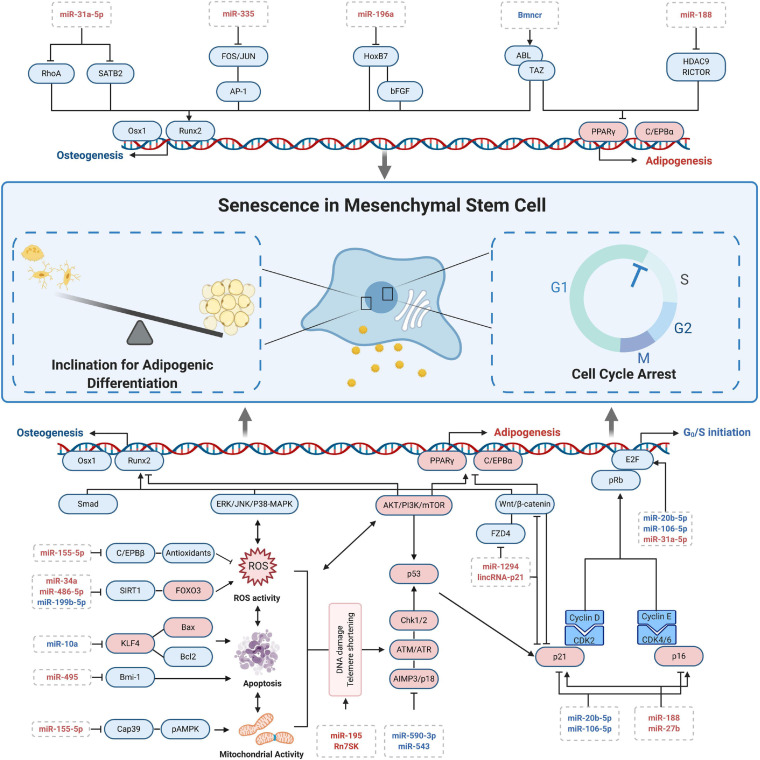
MicroRNA-related regulation at cellular senescence of MSCs.

MicroRNAs’ role in CS is a recent hot spot of research given that advanced techniques like microarray sequencing and bioinformatics are widely utilized. With these techniques, miRNAs were revealed to have an extensive range of gene control. To function, they would bind to the 3′-UTR of target genes involved in DNA damage, epigenetic changes, and metabolism to induce RNA degradation and/or repress translation (Choi et al., 2017). In this part, we mainly conclude the mechanism as well as possible application of miRNAs in senescence-related processes of MSCs (Figure 2).

### Balancing Lever of MSCs’ Fate Against CS, Inflammation, and Apoptosis

Excessive ROS are produced when the imbalance occurs between the free radicals’ generation and scavenging, usually induced by hypoxia. While moderate ROS would ensure the regular biological processes, superfluous ones can lead to DDR and p53 pathway activation, thereby causing cellular apoptosis or senescence (Acín-Pérez et al., 2014; Yee et al., 2014). Prominently, miR-155-5p appears to be a direct connector of ROS generation, inflammation, and aging process in MSCs. The rise of miR-155-5p in the context of aging or aging-related inflammation would target the master transcription factor C/ebpβ to down-regulate a set of antioxidants in ROS generation like superoxide dismutase (SOD), hemeoxygenase-1 (HO-1), and nuclear factor erythroid 2-related factor 2 (NFE2L2). Excessive ROS is produced consequently, hence activating stress kinases and leading to cellular dysfunction (Onodera et al., 2017). P53, as the key mediator of ROS-induced activities, is regulated by an intricate network to ensure the delicate decision of cell fate under hypoxic circumstances. As a most prominent miRNA involved in CS and tumorigenesis, miR-34a contributes to the feedback regulatory loop connecting critical factors like PTEN, SIRT1, FOXO3a, and p53 as mentioned above. In MSCs, over-expression of miR-34a powers the ROS-induced senescence via regulation of the SIRT1/FOXO3a pathway, which is proposed to modulate the anti-oxidative protective effects in diversified tissues (Yamakuchi et al., 2008; Lai et al., 2013; Mouchiroud et al., 2013). Excessive miR-34a would trap SIRT1 to abolish the suppression effect on FOXO3a, increasing the ROS activities (Brunet et al., 2004; Zhang et al., 2015). Another miRNA that shows its inhibition role on SIRT1 deacetylase activity in AD-MSCs is miR-486-5p. In particular, miR-486-5p, rather than miR-34a, would respond to the high glucose changes and result in SIRT1 down-regulation to induce replicative senescence, suggesting its unique role in metabolic disorders (Kim et al., 2012). Additionally, another miRNA predicted to target at SIRT1, miR-199b-5p, showed a negative correlation with the aging process in RNA-seq; however, the mechanism was still open for exploring (Peffers et al., 2016). As a key member of a well-recognized senescence-related miRNA group called miR-17/92 cluster as aforementioned, miR-17 promises to be the linker of inflammation and aging in MSCs as well. It was suggested to bridge the effect of p53 with senescence by targeting Smad ubiquitin regulatory factor 1 (Smurf1) (Liu et al., 2015). P53 would dampen the transcription of miR-17/92 cluster and offset miR-17 inhibition on Smurf1, resulting in senescence and impaired osteogenesis. Notably, though miR-17 was reported to bind transcription factor 3 (TCF3) and Smurf1 to block their negative regulation on osteogenesis, in CS, it is Smurf1 that showed a fluctuating expression level while TCF3 remained unaffected by any treatment of p53 or miR-17. In previous studies, binding of miR-17 to TCF3 and Smurf1 was illustrated to have higher specificity in the normal and inflammatory microenvironment, respectively (Liu et al., 2011, 2013). Considering that inflammatory molecules tend to have elevated expression profiles in senescent cells, it may indicate that the microenvironment would possibly alter the function of miR-17 involved.

**FIGURE 3:**
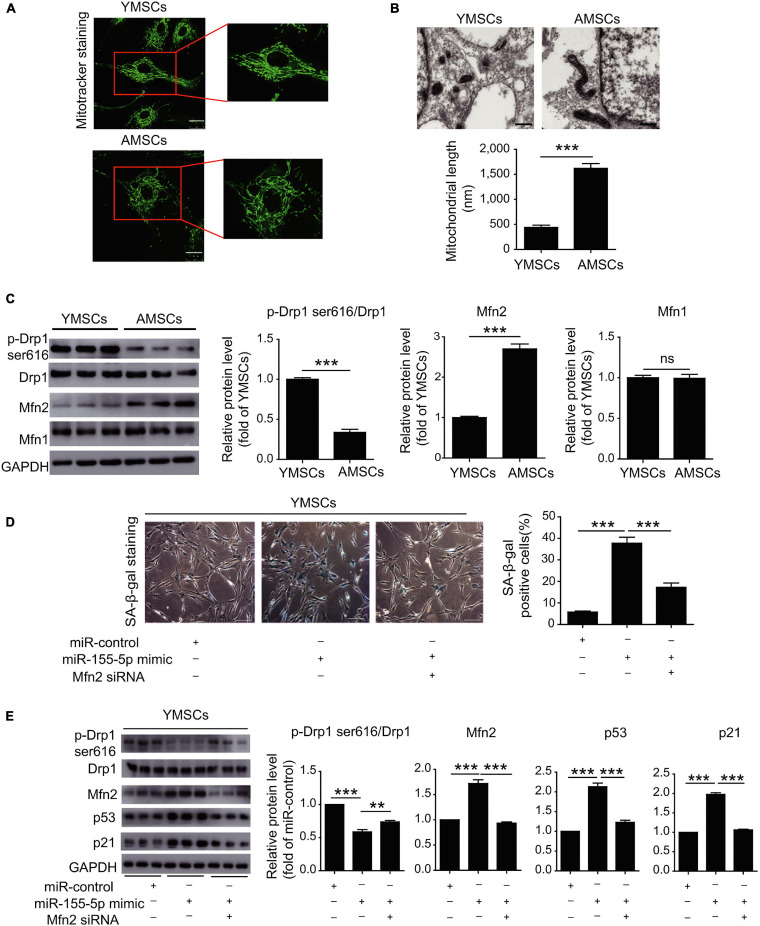
MiR-155-5p inhibition rejuvenates aged mesenchymal stem cells and enhances cardioprotection following infarction. **(A)** MitoTracker staining of YMSCs and AMSCs. **(B)** Mitochondria in ECM and mitochondrial length in AMSCs and YMSCs. **(C)** Expression levels of p-Drp1 (Ser616), Mfn1 and Mfn2 in AMSCs and YMSCs. **(D)** SA-β-gal staining and quantitative analysis of SA-β-gal-positive YMSCs transfected with miR-control, miR-155-5p mimic, or miR-155-5p mimic + Mfn2-siRNA. **(E)** Expression levels of p-Drp1 (Ser616), Mfn1 and Mfn2 in YMSCs transfected with miR-control, miR-155-5p mimic, or miR-155-5p mimic + Mfn2-siRNA. YMSC, yound MSC; AMSC, aged MSC; Scale bar: **(A)** 25 μm **(B)** 500 nm **(D)** 200 μm. Data are expressed as the mean ± SEM. *n* = 3. **p < 0.01; ***p < 0.001. ns, not significant. Reprinted from Hong et al. (2020).

As for the fate of stem cells against hypoxia context, the intricate balance is also determined by manifold factors like autophagy and mitochondrial activities. Stem cells would take autophagy as a protective strategy to remove cellular detriments before being overwhelmed and avoid death or CS (Rastaldo et al., 2020). Aminoacyl-transfer RNA synthetase-interacting multifunctional protein-3 (AIMP3)/p18, as a crucial regulator of autophagy-associated anti-aging mechanisms in MSCs, would dissociate from the multi-tRNA synthetase complex and relocate in the nucleus to enable its DNA restorative ability in an ataxia telangiectasia mutated protein (ATM)/ataxia telangiectasia and Rad3-related protein (ATR)-p53-mediated way (Bensaad et al., 2006; Kim et al., 2011, 2019). Under hypoxic circumstances, the autophagy-involved cellular homeostasis against senescence can be realized by repressed AIMP3, whose induction is inhibited by hypoxia-inducible factor 1α (HIF1α) and promoted by Notch3 (Kim et al., 2019). In young MSCs, miR-543 and miR-590-3p would exhibit their negative impact on senescence by binding the transcript of AIMP3 while the protective effect faded as the descending expression of these two miRNAs was observed in aged MSCs (Lee et al., 2014). It could be speculated that these two miRNAs may serve as anti-aging promoters in an autophagy-related way, which needs further validation. From another aspect, miR-155-5p would regulate senescence by exacerbating the mitochondrial turbulence besides its role in regulating antioxidants as mentioned above (Onodera et al., 2017; Hong et al., 2020). The balance of mitochondrial dynamics between fission and fusion is essential for cell physiology while the imbalance would drive CS (Nishimura et al., 2018; Rizza et al., 2018). Larger tubular mitochondria, indicating higher mitochondrial fusion activity, was observed in late passage MSCs, which may account for replicative CS (Figure 3). Over-expression of miR-155-5p in senescent MSCs would inhibit calcium-binding protein 39 (Cab39)/AMP-activated protein kinase (AMPK) pathway and repressed miR-155-5p in mice was verified to mitigate the mitochondrial fusion, thus rejuvenating aged MSCs and enhancing MSCs therapy effect in cardiac tissue protection (Hong et al., 2020).

Clinically, ischemia of myocardial and neuronal tissue is characterized by accumulation of ROS, which dents the therapeutic effect and accelerates the senescence of engrafted MSCs. MiRNAs mastering ROS activities of MSCs would be therapeutic targets to modulate MSCs for higher survival rates and better functionality in ischemic diseases. Rejuvenation and decreased hypoxia-induced cell apoptosis were observed in aged human BM-MSCs when researchers reversed the descending level of miR-10a artificially. Elevated miR-10a would inhibit the Krüppel-like factor 4 (KLF4)-BCL-2-associated X Protein (BAX)/BCL2 pathway, causing AKT activation to hamper the apoptotic process in senescent MSCs, which would enable the secretion of more angiogenic factors like vascular endothelial growth factor (VEGF) and stromal cell-derived factor (SDF) to exert a therapeutic role in infarcted mouse hearts (Li et al., 2013). As for MSCs under H_2_O_2_ exposure, transfection of miR-34a inhibitor dramatically lowered the death rate by 50%, which stood for a hopeful strategy for MSC transplantation in hypoxic circumstances (Liu et al., 2018). Intriguingly, modified MSCs can deliver miR-34a to glioma cells to realize a therapeutic effect such as inducing DNA damages and subsequent senescence via the SIRT1 pathway (Li et al., 2019). Similarly, MSCs with relatively low expression of miR-34a and increasing SIRT1 may rejuvenate H9c2 cells from a state of doxorubicin-induced senescence, allaying the cardiotoxicity of this chemotherapeutic agent (Xia and Hou, 2018). Therapies based on modified MSCs like miRNA manipulation were already greatly explored to exert anti-tumor effects in other tissues, which still have great potential in the future (Okada et al., 2014; Adams et al., 2016).

To conclude, excessive ROS, induced by manifold circumstances such as hypoxia and beyond, accounts for a main initiator for CS, to which miRNAs show high relevance. On one hand, miRNAs enable the ROS-induced effects, while coordinating the balance between CS and apoptosis confronting ROS through autophagy and mitochondria activity in MSCs. On the other hand, manipulating miRNAs in MSCs would be therapeutically efficient to minimize limitation caused by hypoxic circumstances like ischemia, while maximizing the advantage of ROS in cancer treatment. However, to which degree the miRNA manipulation matters in the process, or the evaluation of miRNA therapy applied as an individual treatment itself or an assistant to standing measures, is not yet well indicated.

### Age-Related Switchers of Differentiation and Cell Cycle of MSCs

A most significant phenotype of senescent MSCs is adipogenesis predilection, which was confirmed and considered as a causative factor for several age-related bone and metabolic diseases like osteoporosis in aged individuals (Pignolo et al., 2019). MiRNAs would target directly at cyclins and the like to either impede or expedite the cell cycle exit to realize terminal differentiation. Several miRNAs with altered expression profile in aged and young MSCs seem to govern the balance and direct the inclination of differentiation of MSCs, about which we would discuss here as promising therapeutic targets.

As direct regulators of E2F/Rb pathway, abnormal expression of several miRNAs would induce cell cycle exit. For instance, members of the miR-17 family such as miR-20b-5p and miR-106a-5p display direct regulation on p21, CCND1, and E2F transcription factor 1 (E2F1), forming a four-way system to modulate E2F1 activity in G1/S transition under oxidative stress-induced senescence, which turns out to be protective and halts the senescence in MSCs (Tai et al., 2020). Similarly, miR-31a-5p would bind to the 3′-UTR of E2F transcription factor 2 (E2F2) and lead to a cascade reaction including SAHF recruitment to accelerate CS in BM-MSCs (Xu et al., 2018).

Alongside their direct effects on cell cycle factors like cyclins, the roles of miRNAs cannot be overlooked such as for conducting the age-related pattern of MSCs’ differentiation by regulating downstream pathways. Up-regulated by the Wnt signaling pathway and down-regulated by interferon-γ (IFN-γ), miR-335 functioned to maintain the stemness of hMSCs, while its reduction seemed to be the prerequisite of differentiation in hMSCs (Tomé et al., 2011). Moreover, it showed a positive correlation with natural or induced CS while forced expression of miR-335 was accompanied by early senescence-like alterations in hMSCs. The precise mechanism remains obscure, but its suppression role on the upstream regulators of activator protein-1 (AP-1) components such as FOS/JUN to reduce AP-1 activity would at least partially mediate the process (Shaulian and Karin, 2002; Tomé et al., 2014). As one particular driver for bone tissue generation, miR-196a appeared to have an inverse correlation with proliferation markers in BM-MSCs and turned out to regulate the age-related expression of HoxB7 (Candini et al., 2015). Representing one member of Hox gene family concerning stem cell hierarchy, HoxB7 would regulate the expression of the differentiation driver as a basic fibroblast growth factor (bFGF) in BM-MSCs (Solchaga et al., 2005; Coutu and Galipeau, 2011; Bhatlekar et al., 2018). Notably, HoxB7 possesses great therapeutic application such as to enhance chondrogenesis as well as the subsequent robust osteogenesis even in AD-MSCs, which are originally less prioritized in bone generation compared to BM-MSCs (Brocher et al., 2013; Foppiani et al., 2019). Intricately, besides their direct role in cell cycle illustrated above, miR-31a-5p and miR-188 act as switchers controlling the balance of osteogenesis. Specifically, miR-31a-5p, utilizing exosomes as vehicles, takes a part in osteoblastogenesis inhibition by targeting the special AT-rich sequence binding protein 2 (SATB2) and in osteoclastogenesis promotion by targeting the RhoA pathway in BM-MSCs (Xu et al., 2018). Bone-relevant markers, such as runt-related transcription factor 2 (RUNX2), osterix (OSX), and Dickkopf homolog 1 (DKK1), are also reported to be bona fide targets of miR-31a-5p, indicating its intricate and ambiguous roles in MSCs (Gao et al., 2011; Baglìo et al., 2013; Deng et al., 2013; Lv et al., 2017). MiR-188 would target the 3′-UTR of histone deacetylase 9 (HDAC9) and rapamycin-insensitive companion of mTOR (RICTOR) to suppress their transcription activities. Both HDAC9 and RICTOR were supposed to be suppressors of PPARγ activity as well as blockers of adipogenic differentiation of MSCs (Chatterjee et al., 2011; Chen et al., 2011; Sen et al., 2014). By diminishing their negative effects on adipogenesis, miR-188 would accelerate the aging-related differentiation pattern switching (Li et al., 2015). Concurrently, the positive and negative correlation of miR-1292 with senescence and osteogenesis markers were verified both *in vivo* and *in vitro*. Its roles in MSCs are proved to be at least partially realized by directly targeting frizzled class receptor 4 (FZD4) and suppressing Wnt/β-catenin signaling (Fan et al., 2018). The inhibition of FZD4 expression on the cell membrane would block its signal transduction from binding Wnt protein to inhibit cytoplasmic glycogen synthase kinase 3 (GSK3) activity, which would accelerate β-catenin degradation. Consequently, accumulation and translocation of β-catenin to the nucleus would facilitate the transcription activities of senescence and osteogenesis-associated genes.

Considering its multipotency, MSC-based tissue engineering accounts for a large proportion of its attraction to researchers, which demands precise control of the differentiation process in MSCs. Thus, age-biased fate of MSCs stands as a hindrance and a challenge for biomedical application. Since evidence of miRNAs playing as age-related switchers of MSC differentiation piles, several indications should be noticed. First, are these miRNAs the cause of altered phenotype or just one of the downstream effectors? Second, when we manipulate these fate-related miRNAs, will other significant phenotypes in tissue engineering such as ability of migration and cell viability be influenced? Third, among all these miRNAs, which one should be the main target to realize our therapeutic goals? Additionally, several miRNAs also turn out to be senescence-related factors in diversified tissue via various pathways. For instance, miR-29 family is widely studied and thought to be a senescence promoter in various tissues. Apart from the significant accumulation of miR-29b-3p in BM-MSC-EVs from aged mice, which would be discussed later, gradual increase of miR-29c-3p in sequentially passaged hMSCs was also verified *in vitro*. Mechanically, miR-29c-3p would promote CS through both p53/p21 and p16/pRB pathways, targeting CNOT6, the subunit 6 of the CCR4–NOT transcription complex, which prevents cell death and senescence (Mittal et al., 2011; Shang et al., 2016). Alongside that, miR-29, together with miR-30, could bridge the inhibitory effect of Rb to cell cycle controller B-Myb post-transcriptionally, while B-Myb could also be suppressed at the transcriptional level by Rb/E2F (Martinez et al., 2011). Besides, miR-29/Ppm1d/p53 constitutes an aging-related circuit in several cellular systems, enabling a universal regulation on DDR and CS (Ugalde et al., 2011). The general crosstalk among ncRNAs like miR-29, miR-34, and senescence, which were identified in diversified cells, from one point of view, could indicate their promising application for MSC-based systematic regulation and, from another perspective, could remind us the risks of general alternation even when we aim solely at MSC manipulation. Certainly, as we explore deeper, more prudent and cautious should we be when making a decision.

## LncRNAs and CircRNAs in CS of MSCs

### LncRNAs

Developments in epigenetic research provide us with deeper insight into the pleiotropic and indispensable functions of lncRNAs, which were once misunderstood as dark matter. With unique modes of functions such as recruiting transcription-associated complexes, regulating epigenetics, and acting as sponges to other complementary RNAs, lncRNA would steer cellular fate in a transcriptional and post-transcriptional way independently or coherently (Chen, 2016). Besides its confirmed key roles in processes like cell proliferation and differentiation in MSCs (Xie et al., 2019), its function in CS was also suggested and ARDs turned out to have a variant expression profile of lncRNAs (Tye et al., 2015; Puvvula, 2019).

Long intergenic non-coding RNA-p21 (lincRNA-p21) correlates with cellular DNA damage and endoplasmic reticulum stress under oxidative conditions (Ning et al., 2015). Responding to DDR, lincRNA-p21 is transcriptionally induced by p53, serving as the scaffold to recruit heterogeneous nuclear ribonucleoprotein-K (hnRNP-K) on the p21 promoter to stimulate transcription regulation (Dimitrova et al., 2014). In MSCs, repressed lincRNA-p21 was proved to enable the process of rejuvenation via the Wnt/β-catenin pathway (Xia et al., 2017). LincRNA-p21 over-expression would reduce the translation of β-catenin and block cytosolic β-catenin’s nuclear translocation, compromising the following effects enabled by Wnt activity (Paige et al., 2010; Brunt et al., 2012). Another ncRNA representing a promising tactic to rejuvenate and maintain the stemness of MSCs is Rn7SK. The Rn7SK small nuclear ribonucleoprotein (Rn7SK snRNP), in which Rn7SK functions as the scaffold to recruit factors like positive transcriptional elongation factor b (p-TEFb), serves as the gatekeeper for RNA polymerase II (Pol II) phosphorylation and activation. Pre-processed AD-MSCs with lower level of Rn7SK showed elevated potential of viability and pluripotency, as well as delayed or halted senescence (Musavi et al., 2019). The protective role is also observed with lncRNA Bmncr to favor osteogenic differentiation of BM-MSCs during aging. Bmncr mainly exerts its function in two aspects, one as niche alteration and the other as promotion of transcriptional complex assembly to switch cell fate of BM-MSCs. Specifically, the specific niche of MSCs plays a decisive role in lineage determination. Variant expression of Bmncr leads to alteration of ECM composition and BM-MSC pool size. Bmncr would affect local 3D chromatin structure and activate the transcription of ECM fibromodulin (FMOD), enabling BM-MSCs anchorage in trabecular-rich metaphyseal regions and promoting the potent osteogenesis inducer–BMP2 pathway in BM-MSCs. Furthermore, its scaffold role facilitates ABL to interact with a transcriptional co-activator with PDZ-binding motif (TAZ). The interaction serves as the catalyst to the successful assembly and activation of the TAZ-RUNX2 and TAZ-PPARγ transcriptional complexes, as the osteogenesis-promoter and adipogenesis-repressor individually, resulting in the switching effect in both mouse and human BM-MSCs (Li et al., 2018). Studies also implied that Bmncr would alleviate osteoporosis by interrupting osteoclast differentiation induced by the receptor activator of nuclear factor-κB ligand (RANKL) (Chen et al., 2019). One promising strategy to deliver exogenous Bmncr or TAZ specifically into BM-MSCs for senile osteoporosis treatment is proposed and was verified to be efficient in animals (Li et al., 2018). However, the study also did not provide us with evaluation of which strategy, such as modifying ncRNA or mRNA alone, or a combination, could be more efficient and promising. Hereto, doubts persist about the strength and drawbacks of ncRNA manipulation, which would be further discussed below.

**FIGURE 4:**
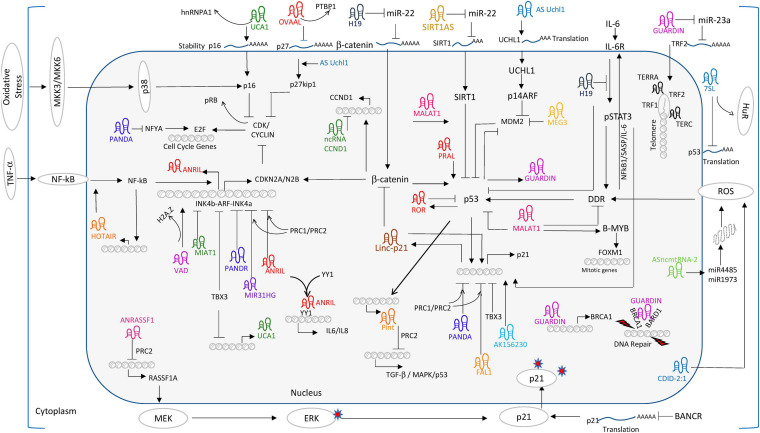
Long non-coding RNA world of regulating CS. Reprinted from Puvvula (2019).

The panorama of senescence-related lncRNAs in MSCs is far from complete. According to a recent review illustrating lncRNA regulatory networks in CS among diversified cellular systems, a number of potential molecules could be introduced for future exploration in MSCs (Puvvula, 2019). Factors and pathways showing high participation in senescence and conserved among species are recommended for research. For instance, Rb/p16 related lncRNAs like very long RNA antisense to dimethylarginine (VAD), myocardial infarction-associated transcript (MIAT), nuclear lncRNA-MIR31 host gene (MIR31HG), antisense non-coding RNA in the INK4 locus (ANRIL), and promoter of CDKN1A antisense DNA damage activated RNA (PANDAR) would lower the level of cyclins to alter E2F-mediated regulation and halt cell cycle at G1-S transition. From another aspect, a set of lncRNAs like p21-associated ncRNA DNA damage activated (PANDA), maternally expressed gene 3 (MEG3), and p53 induced non-coding transcript (Pint) all function in a p53/p21-dependent way, indicating that they may alter the CS process similar to lincRNA-p21 as mentioned above (Xia et al., 2017). Though lncRNAs tend to show high specificity regarding organism and status, existing hints would accelerate the pace to unveil lncRNA network in the senescence process of MSCs (Figure 4).

### CircRNAs

Circular RNAs, generally produced through back-splicing, are characterized by a special covalent loop without 5′ cap or 3′ tail and show resistance to RNase R digestion (Mumtaz et al., 2020). Meanwhile, it possesses highly conservative properties and specificity in expression patterns among cells, tissues, and developmental stages, suggesting its promising role as markers of states like CS. The biological functions are realized through transcriptional and post-transcriptional mechanisms such as alternative splicing, miRNA sponges, and even protein synthesis. Studies suggest a ubiquitous accumulation of circRNAs along the aging process as well as their special expression pattern in aging-related diseases (Maiese, 2016; Cai et al., 2019). Though the significance of overall elevation of expression should be taken with a pinch of salt, considering the higher stability of circRNA during the life period compared with its linear form, it is advisable to view circRNAs as crucial factors in the aging-related process. In MSCs, circRNAs are well-proven to function as important checkpoints during the osteogenesis (Huang et al., 2019b), as well as the gatekeeper for pluripotency maintenance (Cherubini et al., 2019), whereas their function in the senescence process of MSCs remains obscure.

**FIGURE 5:**
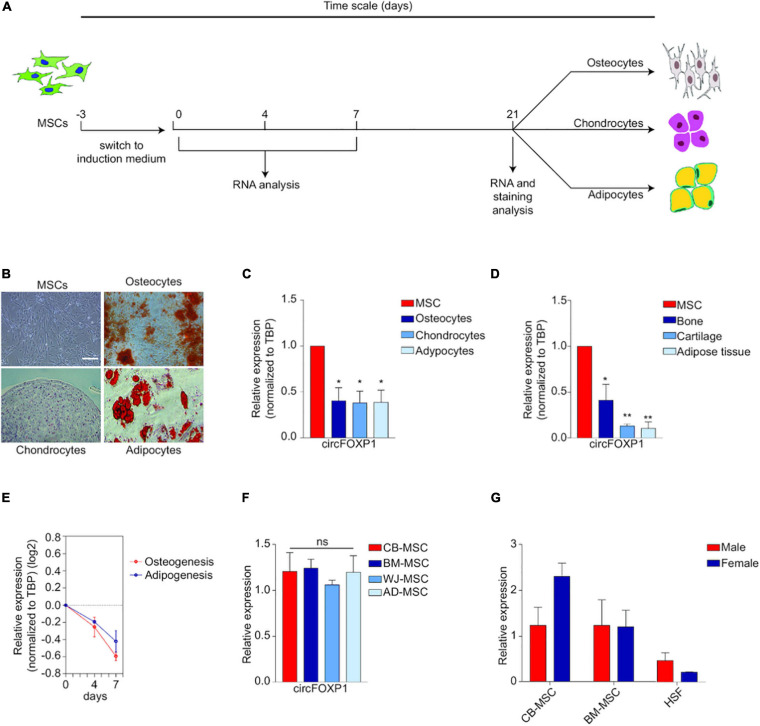
Circ-FOXP1 serves as a specific marker of undifferentiated MSC and are source- and gender-independent. **(A)** MSCs were induced to differentiate into osteocytes, chondrocytes, and adipocytes. **(B)** Images of initiation status and differentiation results of MSCs at 21 days. **(C)** CircFOXP1 expression in different cells. **(D)** CircFOXP1 expression in different tissues. **(E)** The relative transcriptional level of circFOXP1 as MSCs differentiated into different cells. **(F,G)** CircFOXP1 expression in MSCs are source- and gender-independent. WJ-MSCs: Wharton’s jelly MSCs; AD-MSCs, Adipose tissue MSCs; BM-MSCs, Bone marrow MSCs; CB-MSCs, Cord blood MSCs. **(B)** scale bar = 200 μm. Data in **(C,D,F)** are shown as means ± standard error of the mean (*n* = 3); ns, not significant; *P < 0.05, **P <0.01. Reprinted from Cherubini et al. (2019).

In particular, two circRNAs could provide us with a glimpse into the largely unexplored field. In MSCs, hsa_circ_0001320, also named after its linear form as circFOXP1, is borne out to regulate MSCs’ identities independently of the FOXP1 mRNA. CircFOXP1 appears to be more abundant in undifferentiated MSCs compared to all forms of MSC deviates, and circFOXP1 knockdown would dramatically decrease the differentiation capacity of MSCs *in vitro*. Abundant circFOXP1 would act as a sponge to offset the suppression effect of miR17-3p/miR127-5p on non-canonical Wnt pathway as well as active epidermal growth factor receptor (EGFR) signaling (Cherubini et al., 2019). It should be noted that adequate activation of both non-canonical Wnt and EGFR is viewed as one premise for multi-potency maintenance of MSCs (Bilkovski et al., 2010). All these evidences indicate that circFOXP1, as a potent gatekeeper of the multipotent state of MSCs, would be utilized in therapeutic MSCs culturing and manipulating (Figure 5).

Another potential circRNA such as circFOXO3 showed a positive correlation with senescence markers in mouse hearts, while ectopic expression of circFOXO3 in cytoplasm would bind anti-senescence proteins *ID1* and E2F1, as well as anti-stress proteins FAK and HIF1α. The binding would stagnate these four transcription factors from nucleus location, offsetting their anti-senescent regulation functions. The research group also found that circFOXO3 would enhance the interaction between CDK2 and p21, forming a circFOXO3/p21/CDK2 ternary complex. Hijacked CDK2 would lose its ability to trigger DNA replication and S phase transition, blocking the cell cycle progression (Du et al., 2016). Though there is no direct evidence of circFOXO3′s role in MSCs, the FOXO3a protein was reported to reduce senescence by enhancing anti-oxidant enzyme activities and reducing ROS in MSCs (Chang et al., 2017). Considering its involvement in the classic p21 pathway in senescence, a high possibility of its role in MCSs could be suggested.

Fortunately, studies of circRNAs in aging-related pathways of MSCs turn out to be manifold. CircRNAs involved in pathways aforementioned can be considered as hints for further study in senescence. CircRNA_33287 was proved to block miR-214-3p interaction with Runx3, thus promoting osteogenesis and stimulating ectopic bone formation (Peng et al., 2019). Circ_009056 targeting at miR-22-3p/BMP7 is borne out to be an osteogenesis enhancer as well (Wu et al., 2018). Circ-IGSF11 functioning via miR-199b-5p/GSK-3β axil (Zhao et al., 2016; Zhang et al., 2019) and circ_0127781 sponging miR-210/activin A receptor type 1b (AcvR1b) axial and/or miR-335/Dkk-1 axial in the Wnt signaling pathway can both retard osteogenesis (Mizuno et al., 2009; Zhang et al., 2011, 2019). Moreover, both circ19142 and circ5846 have been identified to target miR-7067-5p during the BMP2-induced osteogenic differentiation (Qian et al., 2017). All the evidences for circRNAs’ involvements in MSCs’ properties suggest a promising field for investigating their detailed roles in CS in the future.

## Relation Between MSC-Derived EVs and CS

Mesenchymal stem cells are highlighted for clinic therapy considering their properties like relative accessibility, multipotency, and immunosuppressivity. However, disadvantages like the time required for expansion, inconsistent qualities varying with ages or culturing time, and even the possibility to induce cell death or tumorigenesis hinder the direct implantation of MSCs (Trounson and McDonald, 2015; Kim et al., 2020). Recently, EVs isolated from MSCs (MSC-EVs) are at the forefront as promising alternatives for their capacity to mirror the parental cells and convey the molecular components at both short or long distances among organisms. Meanwhile, EVs would minimize the administration effect and show higher safety considering their absence of nuclei or the ability to replicate (Baldari et al., 2017). Considerably, MSCs also turn out to be more efficient in producing exosomes compared to other cellular types and show advantages in curing cardiac, kidney, liver, and brain diseases via EVs (Yeo et al., 2013; Cai et al., 2020). Instead of being homogeneous, EVs can be divided into various categories based on sizes and origination, among which microvesicles (MVs) and exosomes are most hotly discussed. MVs vary from 100 to 500 nm and are produced in stress-related conditions by the budding of the plasma membrane. Exosomes, as around 50–150 nm, originate from the inward budding of endosomal compartment and are packed in the multivesicular bodies (MVBs) (Van Niel et al., 2018). Up to now, techniques based on neither physical nor biologic properties to separate exosomes from MVs are far from satisfying and we would use EVs as a collective name in this review.

When it comes to the relationship between CS and MSC-EVs, the bidirectional impacts should be considered. To begin with, inhibition on the secretion of EVs in either normal or senescent cells would induce ROS-DDR and cell cycle arrest (Takahashi et al., 2017). Elevating secretion and abnormal phenotypes of EVs in aged cells including senescent MSCs were also verified (Hashimoto et al., 2015; Fafián-Labora et al., 2019), which may elucidate a mechanism utilized by cells to eliminate excessive stuff like harmful cytoplasmic DNA as a response to maintain homeostasis (Lehmann et al., 2008). From another aspect, changes of MSCs along with senescence status are well reflected by MSC-EVs as a key factor in SASP (Lei et al., 2017; Terlecki-Zaniewicz et al., 2018), which would modulate tissue homeostasis and induce paradoxical effects according to different context and status (Lunyak et al., 2017; Jafarinia et al., 2020). To be specific, SASP is used to term the secretion of molecules like growth factors, cytokines/chemokines, and extracellular matrix remodeling enzymes in a senescence-dependent manner, which would extensively in turn impact the senescent process in an autocrine or/and paracrine way (Lunyak et al., 2017). Remarkably, ncRNAs were valid to be enriched in EVs, which appear to carry the majority of miRNAs in circulation (Jafarinia et al., 2020). As for the senescent state, miRNAs in EVs were verified to be a key component of SASP and packaged in a more active mode as information delivery, other than a form as passive garbage disposal (Terlecki-Zaniewicz et al., 2018). Meanwhile, a latest study confirmed that differentially expressed miRNAs displayed a sharper alternation in MSC-MVs than in MSCs, among which MV encapsulated miR-146a-5p holds the potential to be seen as a biomarker for senescence monitoring (Lei et al., 2017). Studies were carried out to elucidate the crucial role of SASP-related miRNAs such as spreading senescence signaling and synchronizing the aging process systematically (Lunyak et al., 2017; Panda et al., 2017). Thus, as potent epigenetic modulators and strong regulators of the microenvironment, ncRNAs in senescence-related MSC-EVs are worthy to be explored and discussed.

### EVs Serve as a Messenger to Synchronize Senescence-Related Process

As mentioned above, co-culture of MSCs from late passages with those from younger ones would induce coherent senescence, where EVs may serve as the explanation. Studies show that age-related MSC-EVs would modify the performance of both parental and younger cells in the niche through an autocrine way (Severino et al., 2013). For instance, elevated miR-183 cluster (miR-96/-182/-183) was observed in aged MSC-EVs, and administration of EVs abundant in miR-183-5p would in turn inhibit osteogenesis and induce senescence in younger MSCs. Mechanically, excessive miR-183-5p transported via EVs would target HO-1 to diminish the anti-oxidative effect, promoting senescence in younger cells (Davis et al., 2017). In a larger picture, CS in MSCs would also act as the initiator to induce systematic changes via EVs. As latest researches attach great importance on systematic pathology and therapies, bone is now viewed as an endocrine organ to regulate metabolism of the whole body (Suchacki et al., 2017). The abundance of miR-29b-3p in BM-MSC-EVs of aged mice could contribute to insulin resistance in aged individuals via targeting at SIRT1. The EVs could transmit the senescence information from BM-MSCs to liver, muscle, and adipose tissue, decreasing their insulin-stimulated glucose uptake. Notably, the clinical application seems to be promising, considering the achieved success in ameliorating insulin resistance of aged mice by delivering modified exosomes containing lower miR-29b-3p (Su et al., 2019).

**FIGURE 6:**
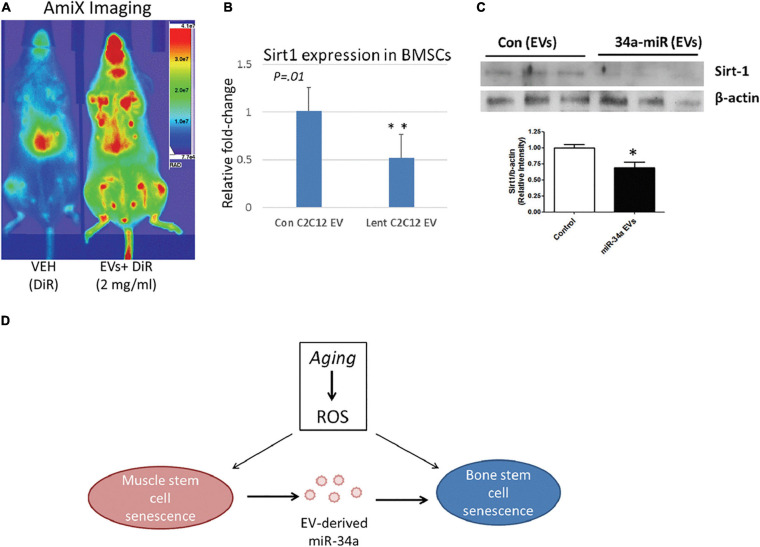
MiR-34a containing EVs home to bone marrow *in vivo* and reduce SIRT1 expression *ex vivo*. **(A)** Mice receiving labeled EVs show high image intensity in the metaphyseal regions of long bones. **(B,C)** Co-culture with EVs from miR-34a overexpression cells reduced Sirt1 expression in BMSCs. **(D)** Role of EV-derived miR-34a in muscle and bone senescence. ROS, reactive oxygen species. *P < 0.05 and **P < 0.01. Reprinted from Fulzele et al. (2019).

Vice versa, ncRNAs secreted by different cell types going through CS would also circulate with EVs, which enable their uptake by MSCs for their aging-related function to play. For instance, exposure of MSCs to senescent EVs separated from blood plasma of elderly donors would lower the osteogenic ability of MSCs, and increased miR-31 turned out to be the crucial connector. Secreted by senescent endothelial cells, miR-31 was packed in MVs to enter MSCs and targeted at Fizzled-3 (FZD3), a Wnt5a receptor, to impair osteoblastogenesis. Consequent fluctuation in the delicate balance between osteoblasts and osteoclasts would finally contribute to unsatisfying quality and quantity of mineral tissues (Weilner et al., 2016). Similarly, elevated miR-34a in muscle-derived EVs from aged mice as well as in EVs isolated from the conditioned medium simulating oxidative stress was verified to contribute to CS initiating in BM-MSCs by targeting Sirt1. Persuasively, the homing ability of EVs containing over-expressed miR-34a to bone marrow was also impaired, and aging-induced ROS accumulation in muscle would alter the physiology of bone (Fulzele et al., 2019; Figure 6). Thus, senescence in other cellular types could trigger the accompanying loss of BM-MSC population and impair bone formation, in which EVs may serve as the messenger for synchronization (Weilner et al., 2016; Fulzele et al., 2019).

### Senescence Put a Strain on the Efficiency of MSC-EV Based Therapy

Apart from inducing the systematic aging process, alteration in senescent MSCs would impair and even offset therapeutic roles of MSC-EVs to heterogeneous cells and organs, which were verified in both chronic and acute diseases in organs like kidney, lung, and spinal cord, as well as inflammatory and metabolic processes (Cai et al., 2020; Varderidou-Minasian and Lorenowicz, 2020). Notably, as MSC-based therapy gains increasing popularity, the changed profile should be highlighted and explored especially when utilizing MSC-EVs extracted from older donors or from late-passage MSCs considering their possible side effects.

Generally, MSC-EVs are largely exploited for their advantageous effects on tissue repair (Varderidou-Minasian and Lorenowicz, 2020). When administered for renal tissue regeneration, MSC-EVs would obstruct epithelial-to-mesenchymal transition (EMT) of the renal tubules through the inhibition of TGF-β1, thus hampering the progress of renal interstitial fibrosis. Nonetheless, failure of repair was observed when administration was carried out with either aged MSC-EVs or miRNA blocker pretreatment. Deeper investigation in rats unveiled the down-regulation of miR-133b-3p and miR-294 in aged BM-MCS-MV to be the cause of impaired effects (Wang et al., 2015). Though there are reports that miR-294 and miR-133 would, respectively, regulate the TGF-β/GSK3 and TGF-β/Smad3 pathway (Duan et al., 2015; Guo et al., 2015), the detailed mechanism of these two miRNAs in this study was not investigated. Besides, three other miRNAs, such as miR-344a, miR-423-3p, and miR-872-3p, also showed significantly lower expression level in aged BM-MSC-MV, though the causes and results of their reduction remain to be explored (Wang et al., 2015).

Apart from regeneration, the immune property of MSC-EVs has also long been tempting for clinical application (Zhou et al., 2019). Nonetheless, the immune-response profile of stem cells turned out to be greatly influenced by senescence (Efimenko et al., 2015; Zeuner et al., 2015). Notably, inflammaging, a new term, was introduced by researchers to name the phenomenon where the Toll-like receptor (TLR) family decreases along with the aging process (Olivieri et al., 2013). Specifically, TLR4 signaling could activate the transcription factor NF-κB and AP-1 to increase inflammatory genes. In MSC-EVs, a significant decrease of several TLR4-related miRNAs, such as miR-21, miR-146a, miR-132, and miR-155, was observed in line with the increase of donor’s age. Showing the most significant changes in accordance with age, miR-21 decreases along with age and may disrupt TLR4-mediated immune response through the AKT/mTOR pathway and up-regulate Wnt5a expression (Fafián-Labora et al., 2017). Conversely, miR-335 in MSC-EVs increases with age, reducing activation of protein kinase D1 (PRKD1) to hamper the transcription activity conducted by AP-1 (Tomé et al., 2011, 2014; Fafián-Labora et al., 2017).

The inducing effect of MSC-EVs on immunosuppressive M2 polarization of macrophages accounts for another crucial mechanism of their anti-inflammatory property (Henao Agudelo et al., 2017; Sicco et al., 2017), whereas it turns out to be eliminated by senescence when applied in diseases like acute lung injury. When isolated from healthy but aged donors and administered to young mice with LPS-induced lung injury, MSC-EVs failed to exert expected protective effects such as inducing M2 polarization or decreasing recruitment. Aside from less internalization of EVs from aged MSCs by macrophages, the failure could also be explained by elevated miR-127-3p and miR-125b-5p in aged MSC-EVs, which would skew macrophages toward the M1 phenotype (Essandoh et al., 2016; Huang et al., 2019a). Controversially, MSC-EVs pretreated with hypoxia (HExos) showed a higher level of miR-125b-5p, followed by a strengthened therapeutic effect on ischemic cardiomyocytes both *in vitro* and *in vivo*, since the miRNA could decrease the expression of target genes such as p53 and BAK1 (Zhu et al., 2018). The discrepancy may be explained by different microenvironments or the synergistic effect between miR-127-3p and miR-125b-5p, which remains to be explored. Interestingly, the M2 shift-inducing ability seems to be also promoted in HExos via altered expression of other ncRNAs. Elevated lncGm37494 targeting miR-130b-3p (Shao et al., 2020), as well as increased miR-216-5p functioning through TLR4/NF-κB/PI3K/AKT cascades (Liu et al., 2020), would both promote recovery effects of HExos on spinal cord injury (SCI) by shifting macrophage polarization toward the M2 phenotype. Intricate interplay among cells enabled by EVs containing ncRNAs merits our attention to achieve desirable tissue repair and immunomodulatory properties.

Evidence piles supporting the idea that EVs serve as a message conveyer to synchronize senescent signals inside and outside MSCs, and ncRNAs embedded could carry the information and initiate CS in recipient cells. Apart from the direct implication of synchronization such as having a systematic view of aging, what should also be reflected on is the impaired ability of regeneration and immunomodulation of EVs derived from aged MSCs. How to take strength of the aging-related phenotype of MSC-EVs and avoid undesirable effects should be tackled, like detecting the senescent markers and abolishing CS-inducing culturing factors. Moreover, MSC-EV-based therapy should be taken and adjusted case to case, considering the alternation caused by environment.

## Therapeutic Potential and Implication

The ultimate goal shared by studies above is to optimize clinical application such as minimizing the undesired senescent properties while maximizing the curing potential of MSC-based therapies. NcRNA-based regulation represents a feasible strategy to achieve that purpose. To be specific, the manipulation is generally realized by exploiting ncRNAs as therapeutic targets and/or tools (Poller et al., 2018). From the first aspect, miRNAs are exploited as endogenous therapeutic targets, which gives birth to strategies like miRNA mimics to store the miRNA activity or anti-miRNAs (antago-miRs) designed to target the miRNAs to be inhibited (Leggio et al., 2017). Similarly, lncRNAs are targeted by tools including RNA interference (RNAi), CRISPR/Cas9, and antisense oligonucleotides (ASOs), for performance improvement (Matsui and Corey, 2017; Micheletti et al., 2017; Neppl et al., 2017). Secondly, artificial ncRNAs like small interfering RNA (siRNA) could induce sequence-specific regulation of target gene expression/translation (Setten et al., 2019), with higher specificity and less off-target effect (Bartoszewski and Sikorski, 2019), which outweighs its counterparts (Yang et al., 2019).

Non-coding RNA therapy strategies targeting MSCs include *in vivo* and *in vitro* approaches. As for *in vivo* therapy, ncRNAs are packaged in vectors and introduced directly into a localized body area to target MSCs, which may be a silver lining for *in situ* MSC rejuvenation. In terms of *ex vivo* approach, MSCs are therapeutically modified by ncRNAs in a laboratory setting and then transplanted (Ocansey et al., 2020). Notably, various ncRNAs could be manipulated toward improving MSCs therapeutic properties via enhanced migration, adhesion, survival, and reduced senescence. For instance, two members of the miR-17 family, such as miR-20b-5p and/or miR-106a-5p mimic-transfected senescent cells, showed significant higher growth rate than controls (Tai et al., 2020). MiR-195 knockdown would reactivate Tert, enabling the telomere re-lengthening, and following reverse of the cell cycle (Okada et al., 2016). Abrogation of excessive lincRNA-p21 would abolish its negative effect on Wnt/β-catenin and then the rejuvenation of aged MSCs (Xia et al., 2017). Furthermore, while senescent MSCs usually lose their pluripotency, ncRNA regulation aiming at improving stemness would as well delay or halt senescence. Notably, the lower level of Rn7SK (Musavi et al., 2019) and the proper abundance of circFOXP1 are both verified to be essential to maintain the multipotent state of MSCs (Bilkovski et al., 2010; Chandra et al., 2013), which suggests a plausible strategy for manipulation. More miRNAs with therapeutical potential were also concluded by X. Zhou et al. in a recent review (Zhou et al., 2020).

As aforementioned, two main goals for MSC modification including improved therapeutical performance in and beyond the skeletal system are set for ncRNA therapy strategies. Inspiring outcomes were already achieved and displayed. Taking senile osteoporosis as an example, the decreased osteogenic differentiation capacity of MSCs could be recovered after the specific delivery of antago-miR-31, showing augmenting osterix expression (McCully et al., 2018). BM-MSC-specific aptamer enabling the precise uptake of antagomiR-188 contributed to substantial outcomes such as increased bone formation in aged mice (Li et al., 2015). Systemically, antagomiR-199a combined with hypoxia-preconditioned MSCs may ameliorate liver injury and further liver regeneration after transplantation (Zhang et al., 2020). In cardiac diseases such as ischemia and the like, both oxidative stress and HG are universally observed as accompanying risk factors, which greatly impair the efficiency of MSCs, due to their potent effect on CS induction. Therapeutical over-expression of miR-10a (Dong et al., 2018) or miR-211 (Hu et al., 2016) in aged MSCs before administration strengthened their repairability to ischemic cardiac tissues. On the contrary, independent inhibition of miR-34a, miR-155-5p, and miR-195 in MSCs all cushion the unfavorable effect of senescence on the efficiency of cardiovascular disease remedy (Okada et al., 2016; Liu et al., 2018; Hong et al., 2020).

**FIGURE 7:**
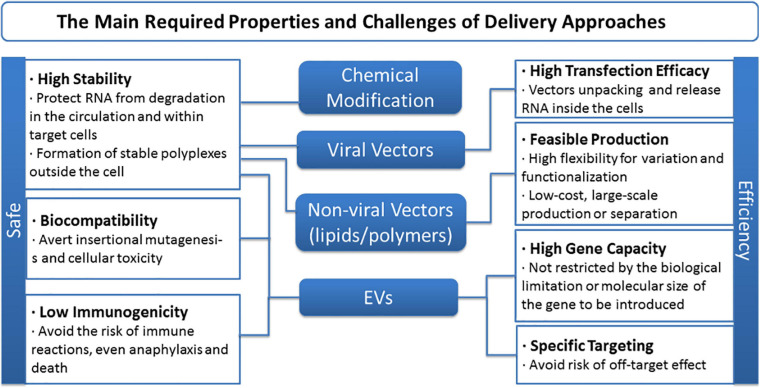
The main required properties and challenges of delivery approaches of non-coding RNA-based therapy.

Although treatment direction is inspiring, drawbacks and challenges of ncRNA-based therapy remain to be contemplated and overcome. First, significant obstacles remain when it comes to naked ncRNA delivery or to enable efficient entrance of any exogenous free RNAs into the systemic circulation. Limited stability and delivery efficiency, for intrinsic flaws such as short half-life, degradation by serum RNases, etc., are all inescapable holdbacks (Egli and Manoharan, 2019; Roberts et al., 2020). RNA editing is one method to boost the stability and potency of miRNA molecule *per se*, while locked nucleic acids (LNA), 20-*O*-methyl modification (20-O-Me) and phosphorothioate backbones are regarded as the most commonly used ones (Nedaeinia et al., 2017). On the other side, the choice of delivery vector strongly influences the efficiency and uptake of nucleic acids in targeted MSCs (Gonzalez-Fernandez et al., 2017). To date, viral and non-viral vectors are generally applied, and the latter can be mainly divided into lipid-based and polymer-based ones. Thanks to the advantages like chemical design flexibility, cationic lipoplexes, PDMAEMA, functional fluorescent vectors, targeting and biodegradable vectors, etc., emerge as competitive tactics (Patil et al., 2019; Scheideler et al., 2020; Wahane et al., 2020). Besides, given their stability and ability to cross biological barriers, EVs open a completely new avenue for therapy that has been illustrated above (Gilligan and Dwyer, 2017; Boriachek et al., 2018). Still, barriers like biocompatibility, gene capacity, and specificity dogged researches, and the main required properties and challenges of delivery approaches of ncRNA-based therapy are concluded in Figure 7. Reasonable design as well as optimization of carrier is the precondition and the following directions might be taken into consideration for better delivery performance: (i) Stability, targeting functions, imaging ability, and biodegradable property need to be incorporated into the development of lipid or polymer vectors; (ii) develop low-cost, large-scale, repeatable isolation technology for EVs and methods for engineering EV content and migration routes; and (iii) apply more elaborate *in vivo* assessments, reducing the current gap between *in vitro* results and *in vivo* performance.

Another challenge is the possibility of off-target effects of ncRNA-based therapy. Considering their size and complexity, synthetic nucleic acid could display unintended binding with proteins and other mRNA targets with partial complementarity (Matsui and Corey, 2017). It is our suggestion that future investigations should consider the following directions: (i) focus more on molecular mechanisms, especially the synergistic roles of ncRNAs, rather than merely observe their physiological effects; (ii) identification of the disease-/tissue-specific functions of ncRNAs to avoid secondary complications; and (iii) multi-drug therapy combining ncRNA therapeutics with other chemical or biological drugs to improve targeting ability (Li and Rana, 2014).

## Conclusion

**FIGURE 8:**
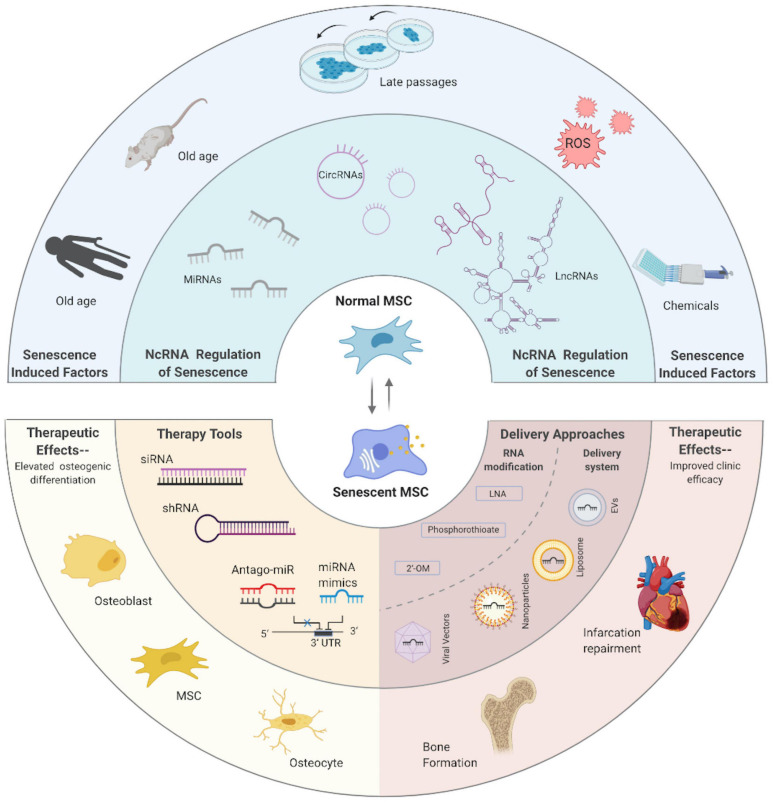
Alternation and manipulation of ncRNAs inside MSCs in a senescence-related way.

As mentioned above, illustrating and impeding the aging process has never been a niche pursuit. Meanwhile, MSCs and MSC-EV-based therapies, though possessing numerous clinical advantages, are greatly hampered by the compromised efficacy and secondary effects that come along with age. Though the final goal to master this seemingly irreversible course remains a longshot, burgeoning progress, especially improvements in the knowledge about ncRNA-related regulation, are quite inspiring and heuristic. Axiomatically, an illustrated map of ncRNAs in the senescent course of MSCs is necessary but still lacking. Thereby, in this review, we display the general background of aging and CS, as well as the relationship between CS and MSCs; then, the intricate ncRNA world of CS is illustrated, including the reported roles of miRNAs, lncRNAs, and circRNAs at play. Furthermore, as MSC-EVs emerge as the potent therapy method, we introduce the ncRNAs embodied in EVs connecting aging and the other process; last, we look into the promising therapeutic fields by modulating ncRNAs in CS of MSCs that hold potential for clinical application. Manifold factors could induce ncRNA alternation, which could either initiate or conduct CS in MSCs, while various strategies could take advantage of ncRNAs to rejuvenate MSCs and achieve better outcomes (Figure 8). Aging in MSCs is easy to detect but hard to prevent, and the attempt to reconcile senescence and rejuvenate stem cells should always be taken with a grain of salt. Re-entry of the cell cycle of MSCs could be oncogenic while aging-inducing effect would also have a good effect when applied in cancer therapy. Deliberate consideration should be made about the regulation network, and our work supports investigation into this process.

## Author Contributions

JC, WL, and ZZ: conceptualization. JC, HQ, KY, DJ, and YY: writing—original draft. JC, HQ, and KY: visualization. WL and ZZ: writing—review and editing, funding acquisition, and supervision. All authors contributed to the article and approved the submitted version.

## Conflict of Interest

The authors declare that the research was conducted in the absence of any commercial or financial relationships that could be construed as a potential conflict of interest.
